# Supplementary material for: Paradoxical Radiosensitizing Effect of Carnosic Acid on B16F10 Metastatic Melanoma Cells: A New Treatment Strategy
Source: Antioxidants (Basel). 2022 Oct 31;11(11):2166. doi: 10.3390/antiox11112166 (PMC9686564; doi:10.3390/antiox11112166)
Supplement: Supplementary file 1 [file antioxidants-11-02166-s001.zip › antioxidants-1960085-supplementary-final.pdf]

antioxidants

MDPI

Optimization of cell culture parameters for cell survival assay.

The determination of the optimum parameters of the cell cultures in the MTT assay was carried out with four (4) initial objectives:

1. Determination of the maximum non-toxic concentration of CA on PNT2 cells i.e. the normal or reference concentration (100% cell survival).
2. Determination of the radiation dose for assessment of radioprotective and/or radiosensitizing effect of CA in the most radiosensitive cells (PNT2) and definition of three important parameters (X-ray dose, CA concentration and cell incubation period).
3. Confirmation of these conditions in B16F10 melanoma cells.
4. Confirmation of the results obtained using different mixtures of other antioxidant substances.

1. Determination of the maximum non-toxic concentration of CA on normal cells (PNT2).

Initially, different concentrations of CA were tested on PNT2 cells to determine its optimum non-cytotoxic concentration on cell survival (Figure S1).

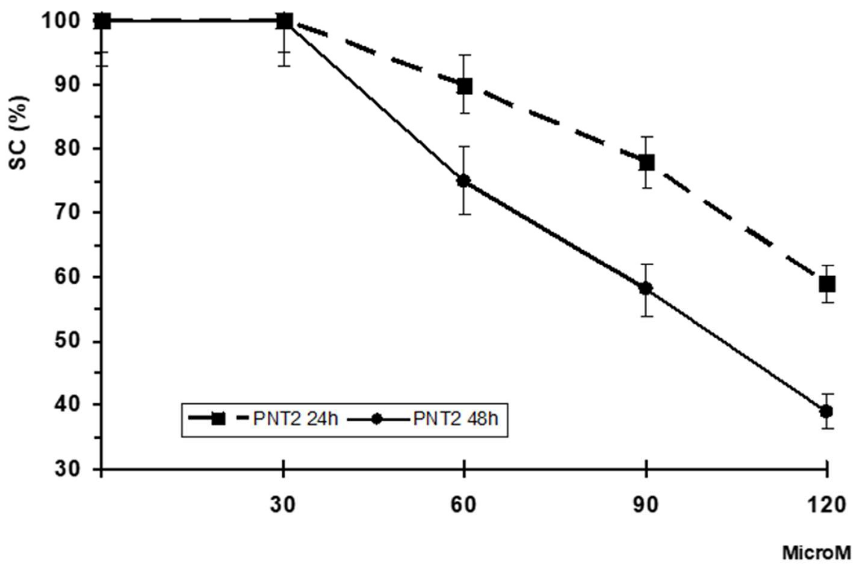

Figure S1. Effect of different concentrations of CA on PNT2 cell survival.

In this study, the inhibition of cell growth in PNT2 was noted at a concentration of 30 μM. Therefore, two concentrations within the range that produced 100% cell survival were retested: 20 and 40 μM. The 20 μM solution did not portray any significant toxic effect on PNT2 cells at any of the two incubation periods tested (Figure S2).

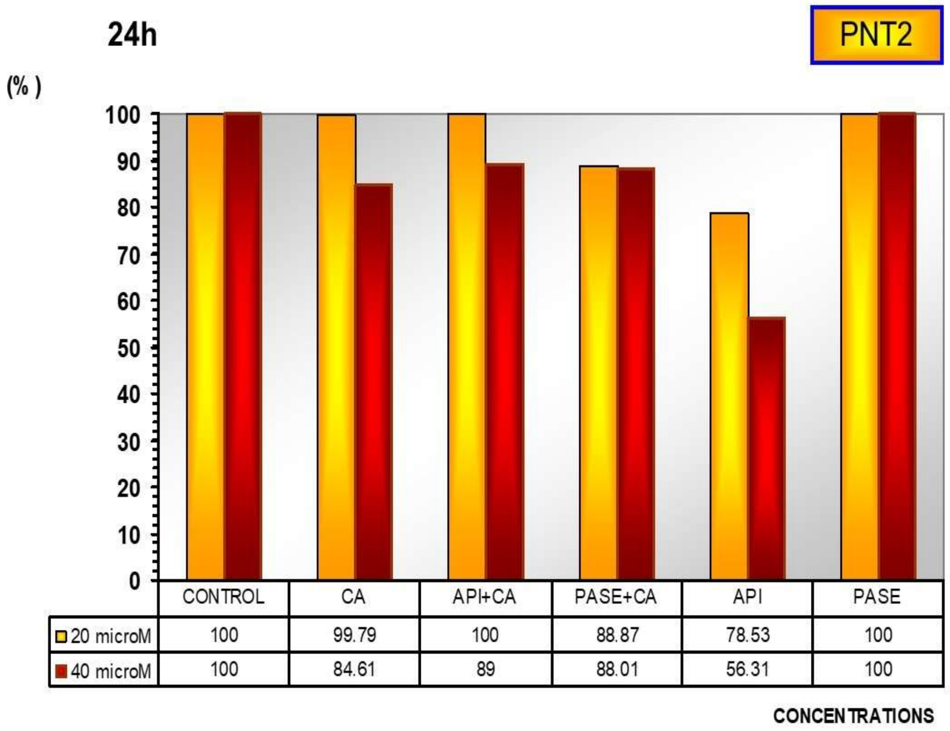

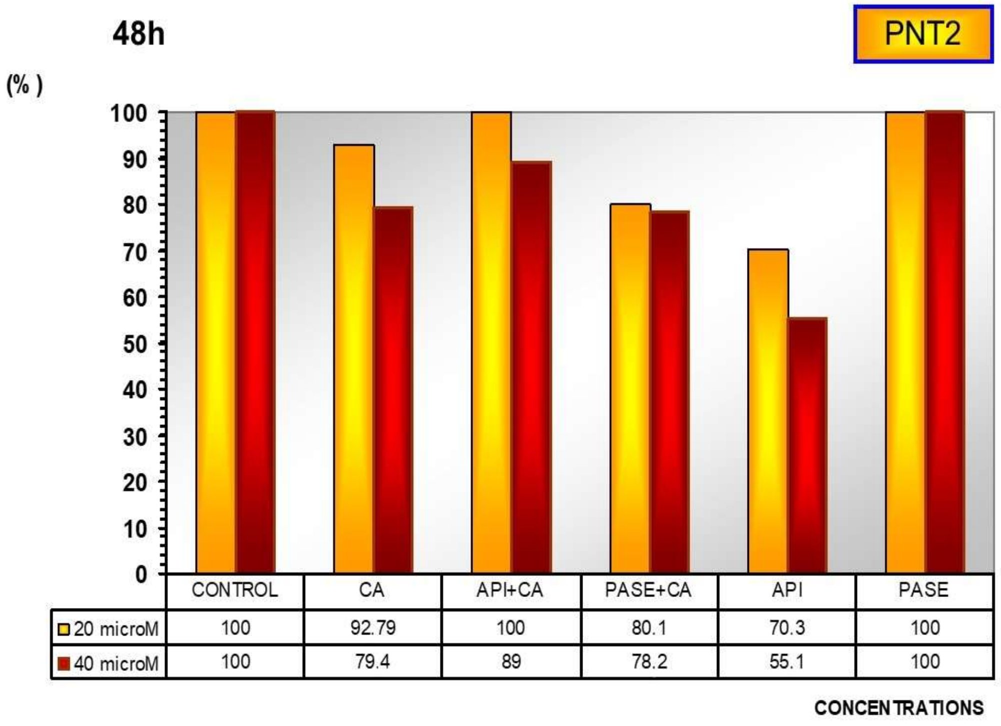

Figure S2. Percentage cell survival of control cell cultures after the administration of the different substances studied on PNT2 cells evaluated after a 24 and 48-hour of incubation period (API: Apigenin; CA: Carnosic acid; PASE: Pycnanthus angolensis Warb Seed).

These results shows that the concentration of CA that meets these requirements should be less than 30 μM.

2. Determination of radiation dose for assessment of radioprotective or radiosensitizing effect of CA in the most radiosensitive cells (PNT2) and definition of three important parameters.

a). X-ray doses We initially started with intermediate doses of X-rays (10-22 Gy) to determine the degree of radiosensitivity of PNT2 cells, which are considered sensitive to ionizing radiation. The results showed extreme mortality in the cells at the radiation doses tested (Figure S3).

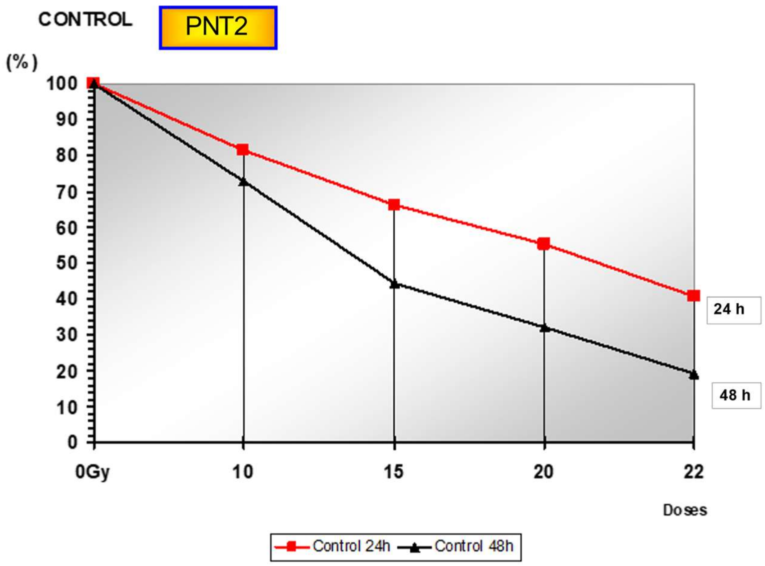

Figure S3. Cellular survival (%) of PNT2 cells irradiated with intermediate doses of X-rays for the two incubation periods tested (24 h and 48 h).

Therefore, the tests were repeated with lower doses of X-rays. The results of analysis conducted on PNT2 cells that were not treated with any substance but exposed to X-ray doses of 0 Gy, 4 Gy, 6 Gy, 8 Gy and 10 Gy are displayed in the table below (Table S1). A progressive dose-related decrease in the number of live cells which varies inversely with the administered radiation dose but directly with the post-irradiation assay period is portrayed i.e., the higher the radiation dose and the longer the post-irradiation incubation period, the lower cell survival. After exposure to 10 Gy of irradiation, the proportion of surviving cells reduced by 30.8% and 38.8% when examined 24 and 48 hours post irradiation respectively (**p<0.001**) (Table S1; Figure S4).

Table S1. Percentage cell survival (%) of normal human prostrate epithelial PNT2 control cell cultures.

Time/Radiation dose	0 Gy	4 Gy	6 Gy	8 Gy	10Gy
24 hours	100.0 ± 5.8	91.0 ± 7.1	86.2 ± 7.2	79.4±5.4	69.2±5.1*
48 hours	100.0 ± 4.5	84.5 ± 8.2	77.3 ± 7.4	71.1±5.6	61.2±5.2*

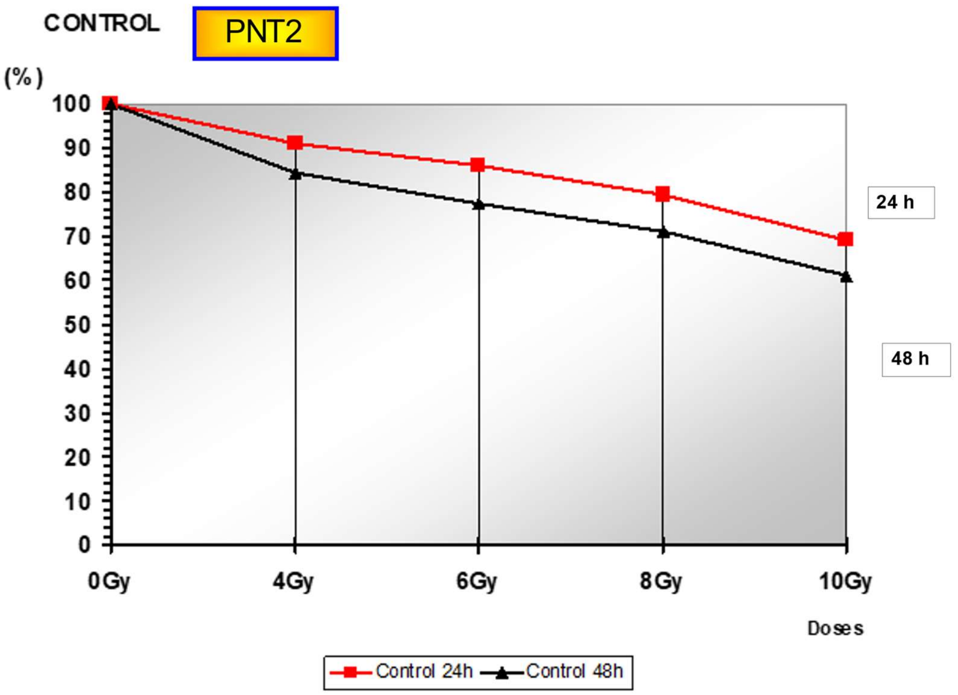

Figure S4. Cell survival (%) curves of irradiated normal human prostrate epithelial PNT2.

These results shows that the radiation dose that registers a harmful effect of X-rays and at the same time could show a protective effect is 10 Gy.

b). CA concentration and cell incubation periods: Due to the selection of a radiation dose of 10 Gy and an incubation period of 48 hours, we tested two different concentrations of CA (10 μM and 20 μM) the effects of which are shown below. In the CA-treated PNT2 cell cultures that were exposed to irradiation and evaluated after 24 hours of incubation, the results obtained from the cell cultures treated with 10μM and 20 μM of carnosic acid portrayed a significantly better cell survival response compared with the irradiated control cell cultures. At the highest radiation dose of 10 Gy used in this study, the proportion of surviving cells in the irradiated control cultures dropped by 30.8% when compared with cell populations in cultures that were sensitized with the two test doses of carnosic acid used. At both concentrations of CA studied, a significant increase in cell survival was shown compared with irradiated control cell cultures ($p<0.001$) (table S2; Figure S5).

Table S2. Percentage cell survival (%) of normal human prostate epithelial PNT2 cell cultures treated with CA (10μM and 20 μM) and exposed to different doses of X-rays evaluated after 24 hours of incubation.

Radiation dose	0 Gy	4 Gy	6 Gy	8 Gy	10 Gy
Control	100.0 ± 5.8	91.0 ± 7.1	86.2 ± 7.2	79.4 ± 5.4	69.2 ± 5.1
10μM Carnosic acid	100.0 ± 8.2	100.0 ± 7.6	100.0 ± 6.9	100.0 ± 7.5	100.0 ± 9.2*
20μM Carnosic acid	100.0 ± 7.9	100.0 ± 8.3	100.0 ± 7.5	100.0 ± 9.0	100.0 ±10.2*

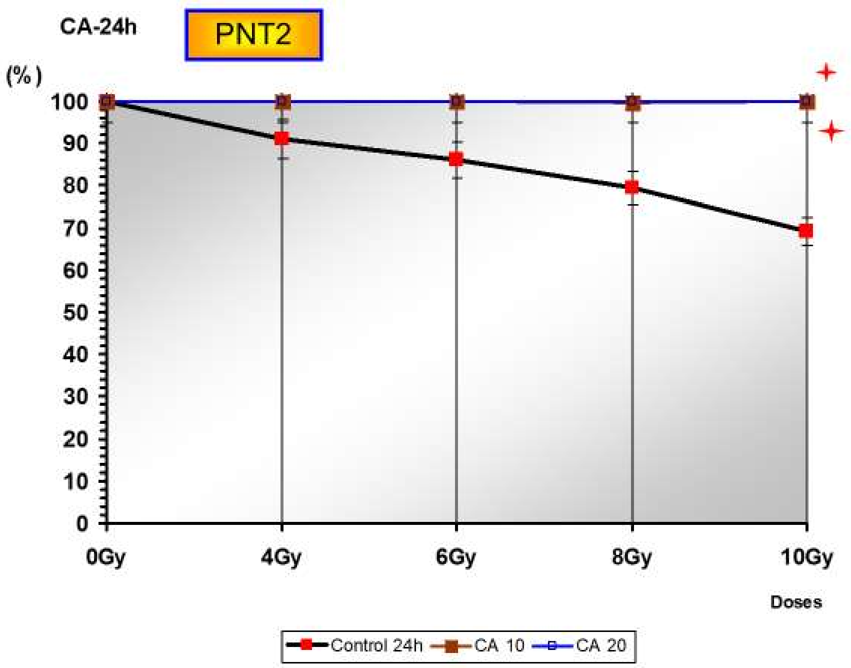

Figure S5. Cell survival (%) curves of normal human prostate epithelial PNT2 cell cultures treated with CA (10μM and 20 μM) and exposed to different doses of X-rays evaluated after 24 hours of incubation. ((*): (p<0.001) versus irradiated control cells).

The **Protection Factor** (**PF**) obtained from the cultures sensitized with carnosic acid and exposed to the highest experimental radiation dose of 10 Gy following 24 hours incubation was 100% for both 10 μM and 20μM of carnosic acid solutions used. After 24hr of incubation, it was not possible to directly obtain the **dose Reduction Factor** (**DRF**) (DRF is the quotient between the radiation dose necessary to produce a given effect in the presence of a radioprotective compound and the radiation dose necessary to produce the same effect in the absence of said radioprotective compound, which makes it possible to determine the reducing/radioprotective capacity of the tested substance, as was described previously [20,36] from experimental results obtained from the cell cultures sensitized with both concentrations of Carnosic acid (10 μM and 20 μM) and exposed to highest radiation dose (10 Gy).

After 48 hours following incubation, the results obtained with PNT2 cell cultures treated with 10 and 20 μM of carnosic acid and exposed to the highest experimental radiation dose of 10 Gy showed a higher rate of surviving cells when compared with the irradiated control cell cultures. At the end of the two incubation periods (24 h and 48 h), cellular survival was equal, standing at 38.8% compared to irradiated controls at this radiation dose. At both concentrations used, a significant increase in PNT2 cell survival was demonstrated compared with irradiated control cell cultures (p<0.001) (Table S3; Figure S5).

Table S3. Percentage cell survival (%) of normal human prostate epithelial PNT2 cell cultures treated with CA (10μM and 20 μM) and exposed to different doses of X-rays evaluated after 48 hours of incubation.

Radiation dose	0 Gy	4 Gy	6 Gy	8 Gy	10 Gy
Control	100.0 ± 4.5	84.5 ± 8.2	77.3 ± 7.4	71.1 ± 5.6	61.2 ± 5.2
10 μM CA	100.0 ± 7.5	100.0 ± 7.4	100.0 ± 9.7	100.0 ± 5.7	100.0 ± 4.9*
20μM CA	100.0 ± 5.7	100.0 ± 9.1	100.0 ± 6.4	100.0 ± 5.7	100.0 ± 5.3*

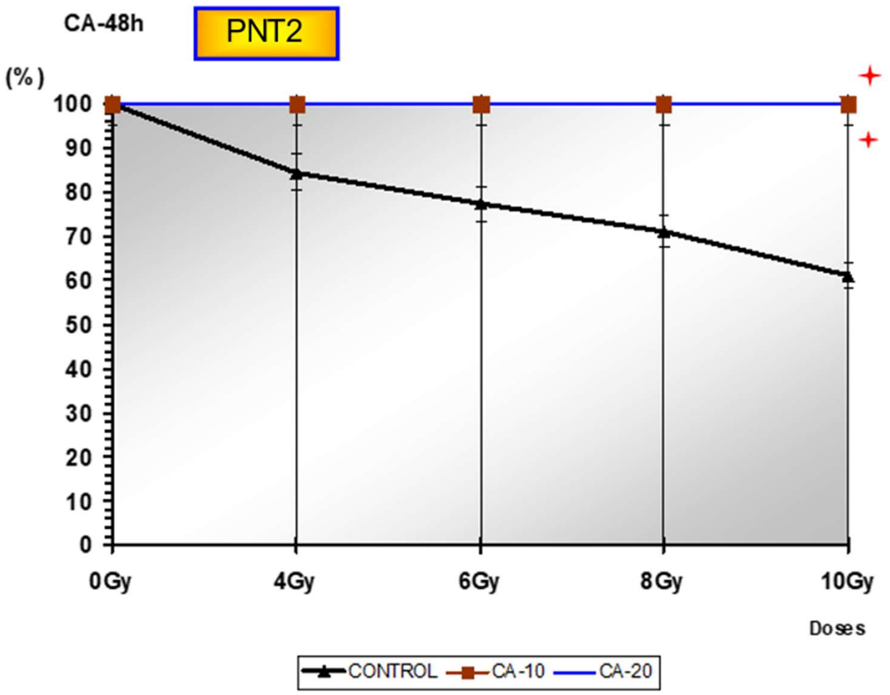

Figure S6. Cell survival (%) curves of normal human prostate epithelial PNT2 cell cultures treated with CA (10μM and 20 μM) and exposed to different doses of X-rays evaluated after 48 hours of incubation. ((*): (p<0.001) versus irradiated control cells).

The **Protection Factor** (**PF**) obtained of the cultures treated with 10 μM and 20μM of carnosic acid following 48 hours and exposure to the highest radiation dose of 10 Gy was 100% for both concentrations of carnosic acid. Upon exposure to the highest radiation dose of 10 Gy, and incubating for 48 h, it was not possible to directly obtain the DRF from the cell cultures exposed to both 10 μM, and 20 μM of carnosic acid.

Under these conditions, we opted to use the maximum concentration of CA (25μM) and the longest incubation period (48h). For the study, we repeated the tests with 25 μM of CA which enabled the use of the maximum radiation dose without modifying cell survival and also allowed us to use the same concentration as employed in the micronucleus test (MNCB).

3. Confirmation of proposed conditions in B16F10 melanoma cells.

Percentage cell survival in B16F10 control cultures after exposure to X-rays

The cell survival results of B16F10 cells not treated and irradiated with various X-rays doses i.e., 0 Gy, 4 Gy, 6 Gy, 8 Gy and 10 Gy showed a progressive decrease in the number of viable cells which varied inversely as the radiation dose and post-irradiation incubation period (**p<0.001**). That is, cell viability decreased with increasing radiation dose and post-irradiation period of incubation; Cell survival after 24 hours incubation reduced by 33.1% at the highest radiation dose of 10 Gy and 41% after 48 hours incubation at the same radiation dose (**p<0.001**) (Table S4; Figure S7).

Table S4. Percentage cell survival (%) of control B16F10 cells cultures.

Time/ Radiation doses	0 Gy	4 Gy	6 Gy	8 Gy	10Gy
24 hours	100 ± 9.5	86.7 ± 7.4	80.6 ±8.2	73.2 ± 6.8	66.9 ± 7.7*
48 hours	100 ± 0	74.7± 8.3	72 ± 6.8	66.0 ± 7.4	59.0 ± 5.9 *

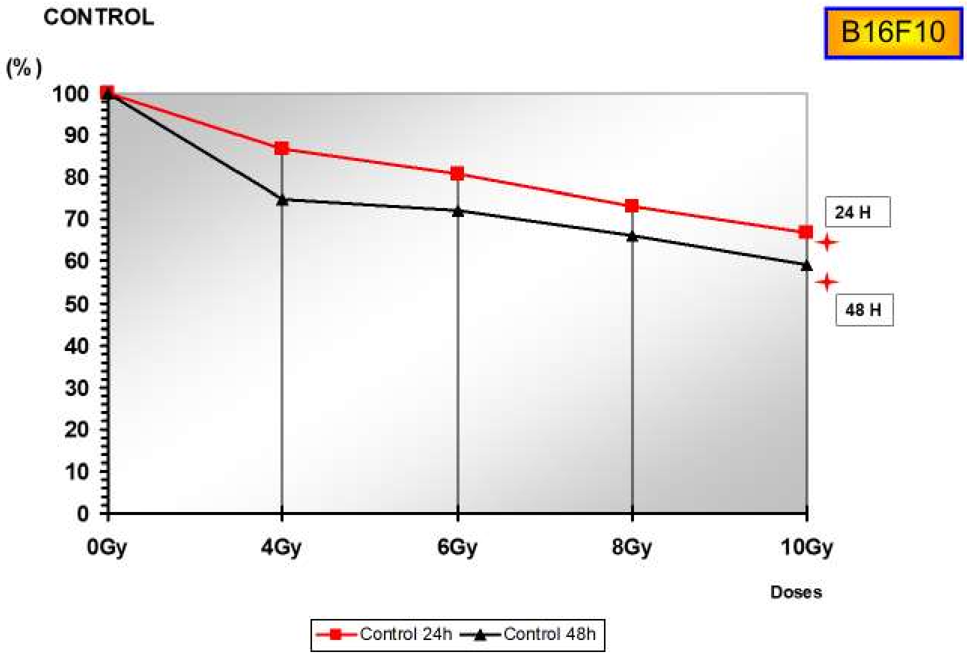

Figure S7. Cell survival (%) curves of metastatic B16F10 melanoma control cell cultures ((*): (p<0.001) versus irradiated control cells.

- CARNOSIC ACID (CA)

After 24 hours of B16F10 cell incubation at both concentrations of carnosic acid (10 and 20 μM) tested, cell survival was observed to increase by 15.5% for the lower concentration (10 μM) and 33.1% for the higher concentration (20 μM) of carnosic acid used when compared with irradiated control cell cultures; in both cases when the highest radiation dose (10 Gy) in this study was used. At both concentrations of CARN used, a significant increase in the survival of B16F10 melanoma cells was established when compared with irradiated control cell cultures (**p<0.001**) (Table S5; Figure S8).

Table S5. Percentage cell survival (%) of B16F10 cell cultures treated with CA (10μM and 20 μM) and exposed to different doses of X-rays evaluated after 24 hours of incubation.

Radiation doses	0 Gy	4 Gy	6 Gy	8 Gy	10Gy
Controls	100.0 ± 9.5	86.7 ± 7.4	80.6 ± 8.2	73.2 ± 6.8	66.9 ± 7.7
Carnosic Acid 10 μM	100.0 ± 8.4	100.0 ± 8.1	100.0 ± 7.6	100.0 ± 7.9	100 ± 10.2*
Carnosic Acid 20 μM	100.0 ± 7.9	100.0 ± 8.4	100.0 ± 8.2	100.0 ± 9.1	82.3 ± 9.2 *

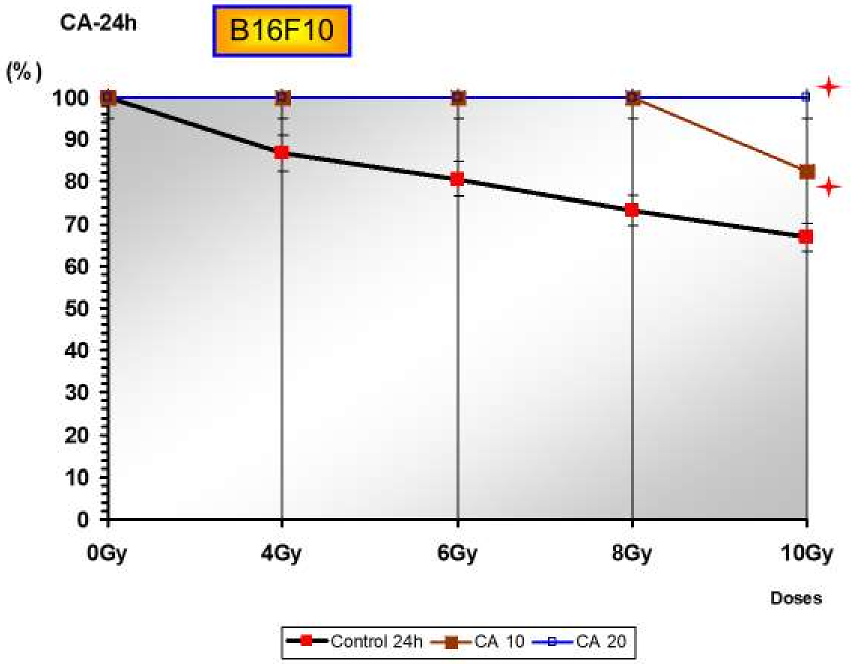

Figure S8. Cell survival curves of B16F10 cell cultures treated with CA (10μM and 20 μM) and exposed to different dosed of X-rays evaluated after 24hours of incubation ((*): (p<0.001) versus irradiated control cell cultures).

Protection Factor (PF) obtained from 24-hour B16F10 cell cultures treated with 10 μM carnosic acid and exposed to the highest dose (10 Gy) was 100% and 46.5% when 20 μM apigenin solution was used. **Dose Reduction Factor (DRF)** obtained for 24-hour cultures treated with 20 μM carnosic acid and exposed to the highest experimental radiation dose (10 Gy) was 1.8 but an equivalent value could not be computed directly from the results obtained with the 10 μM carnosic acid concentration and the radiation dose used.

After 48 hours of B16F10 cell culture following administration of the two carnosic acid concentrations and exposure (10 and 20 μM) to 10 Gy radiation, cell survival decreased by 2.5% and 19.2% compared to controls cell irradiated for the 10 μM and 20 μM solutions respectively. At both concentrations of CA used, there was no significant radioprotective differences when compared with those obtained from the irradiated control cell cultures (**p<0.001**) (Table S6; Figure S9). Contrarily, the administration of CA showed a radiosensitizing effect (Figure S9).

Table S6. Percentage cell survival (%) of B16F10 cell cultures treated with CARN (20μM and 40 μM) and exposed to different doses of X-rays evaluated after 48 hours of incubation.

**Radiation doses**	**0 Gy**	**4 Gy**	**6 Gy**	**8 Gy**	**10Gy**
Controls	100.0 ± 0	74.7 ± 8.3	72.0 ± 6.8	66.0 ± 7.4	59.0 ± 5.9
Carnosic acid 10 μM	100.0 ± 6.9	100.0 ± 7.9	77.7 ± 7.1	62.3 ± 6.2	56.5 ± 6.7
Carnosic acid 20 μM	100.0 ± 8.4	100.0 ± 9.1	76.1 ± 7.4	71.2 ± 6.2	39.8 ±6 $^{\Delta}$

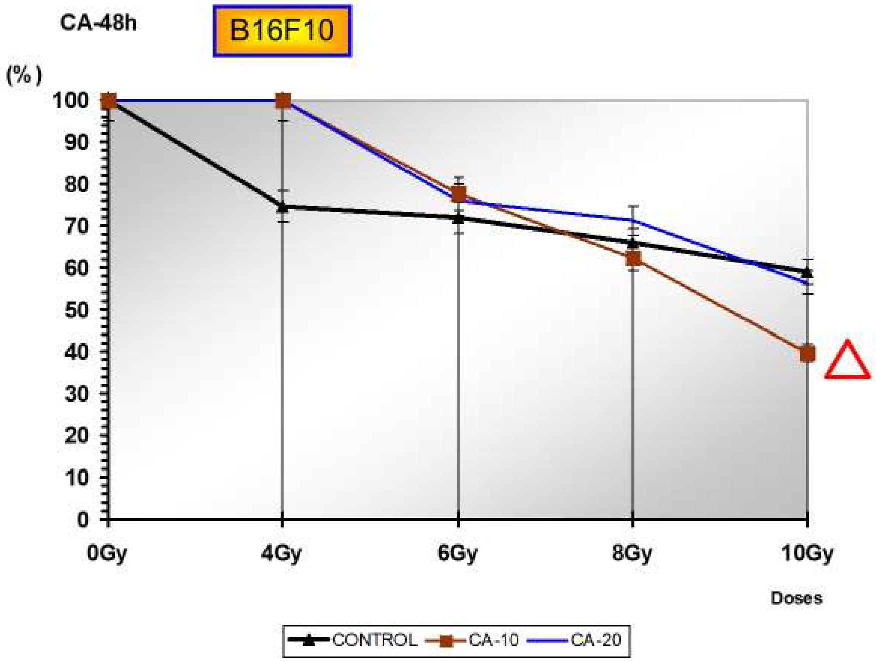

Figure S9. Cell survival curves of B16F10 cell cultures treated with CA (10µM and 20 µM) and exposed to different doses of X-rays evaluated after 48 hours of incubation. ((▲): ($p<0.001$) versus irradiated control cell cultures).

Protection Factor (**PF**) obtained from 48-hour B16F10 cell cultures treated with the two carnosic acid concentrations tested (10 µM and 20 µM) and exposed to the highest radiation dose (10 Gy) was zero, i.e. no radiation protective capacity. It was not possible to compute the **Dose Reduction Factor (DRF**) for 48-hour carnosic acid-treated B16F10 cell cultures. This means that the two concentrations used (10 µM and 20 µM) shows no dose reducing effect.

5. Confirmation of other results obtained using different mixtures and concentrations of other antioxidant substances.

4A) PNT2 Cells

MIXTURES APIGENIN AND CARNOSIC ACID (API +CA)

The simultaneous administration of equimolar amounts of Apigenin and CA i.e., 25 µl of 10 µM (10µM API + 10µM CARN) and 20 µM (µ20 µM API + 20µM CARN) was also evaluated on cell proliferation. After 24-hour incubation, the 10 µM equimolar mixture did not lead to a decrease in the percentage of cell proliferation; however, the 20 µM equimolar mixture elicited an 11% reduction in percentage of surviving cells compared with cell viability values obtained in control cell cultures. Following 48 hours of incubation, there was no decrease in cell survival values at the lower concentration (10µM), but a decrease of 27.5% in percentage cell survival was observed with the 20 µM mixture compared with control cell cultures (Figure S10).

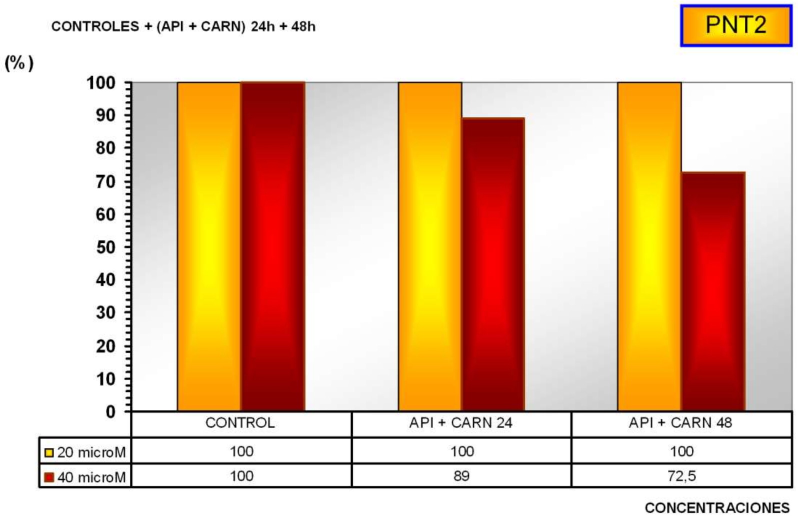

Figure S10. Effect of administration of different concentrations (10μM + 10 μM) and (20μM +20 μM) of (API + CA) mixtures on PNT2 cell survival evaluated after 24h and 48h incubation periods).

After exposure to the highest experimental radiation dose of 10 Gy and incubating cells for 48 hours, the results obtained with the PNT2 cell cultures co-exposed to the two test substances at both concentrations showed a superior cell survival of 27.3% in comparison with the irradiated control cells. In both concentrations, a significant increase in cell survival was demonstrated compared with control cell cultures ($p<0.001$) (Table S7; Figure S11)

Table S7. Percentage cell survival (%) of normal human prostate epithelial PNT2 cell cultures treated with 10 μM and 20 μM mixtures of (API + CA) and exposed to different doses of X-rays evaluated after 24 hours of incubation.

Radiation dose	0 Gy	4 Gy	6 Gy	8 Gy	10 Gy
Control	100.0 ± 5.8	91.0 ± 7.1	86.2 ± 7.2	79.4 ± 5.4	69.2 ± 5.1
10 μM (API + CA)	100.0 ± 7.8	90.0 ± 9.4	90.3 ± 9.8	90.3 ± 7.5	96.5 ± 7.6*
20 μM (API + CA)	100.0 ± 8.1	96.0 ± 7.5	86.2 ± 5.4	97.3 ± 6.9	94.4 ± 8.2*

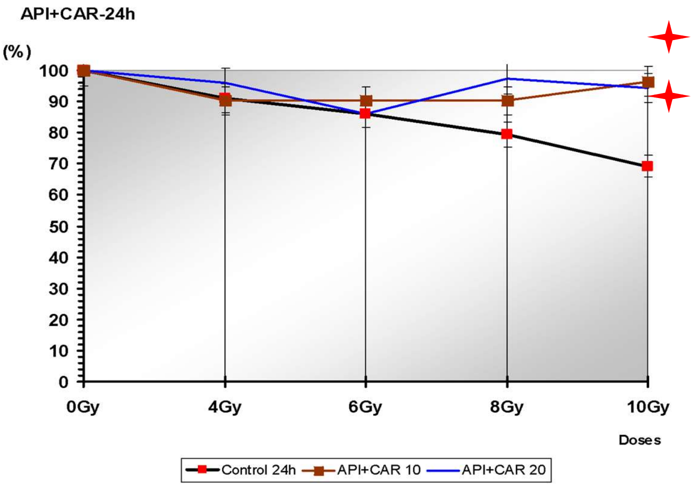

Figure S11. Cell survival (%) of normal human prostate epithelial PNT2 cell cultures treated with 10 μM and 20 μM mixtures of (API + CA) and exposed to different dosed of X-rays evaluated after 24 hours of incubation ((*): p<0.001) versus irradiated control cells).

After exposure to the highest experimental radiation dose of 10 Gy and incubating for 24hours, the PF obtained with the cell cultures treated with the apigenin and CA mixture was 88.6% at equimolar concentration of 10 μM and 81.8% for the 20 μM mixture. When the cell cultures where treated with the highest experimental radiation dose of 10 Gy, and incubated for 48 hours, a DRF of 5.8 was obtained when the cultures were treated with the 10 μM mixture and 3.4 when treated with the 20 μM mixture.

When exposed to the highest experimental radiation dose of 10 Gy following 48 hours of incubation, the PNT2 cell cultures treated with the 10 μM and 20 μM mixtures, showed an increased cell survival of 38.8% for the higher equimolar mixture in relation to the irradiated controls. At both concentrations, a significant increase in surviving cells was demonstrated compared with irradiated control cell cultures (**p<0.001**) (table S8; Figure S12).

Table S8. Percentage cell survival (%) of normal human prostate epithelial PNT2 cell cultures treated with 10 μM and 20 μM mixtures of (API + CA) and exposed to different doses of X-rays evaluated after 48 hours of incubation.

Radiation dose	0 Gy	4 Gy	6 Gy	8 Gy	10 Gy
Control	100.0 ± 4.5	84.5 ± 8.2	77.3 ± 7.4	71.1 ± 5.6	61.2 ± 5.2
10 μM (API + CA)	100.0 ± 8.2	70.5 ± 7.4	70.2 ± 9.1	70.8 ± 7.5	73.2 ± 8.9
20 μM (API + CA)	100.0 ± 7.7	88.5 ± 7.3	90.9 ± 6.5	85.3 ± 2.1	100.0 ±12.4*

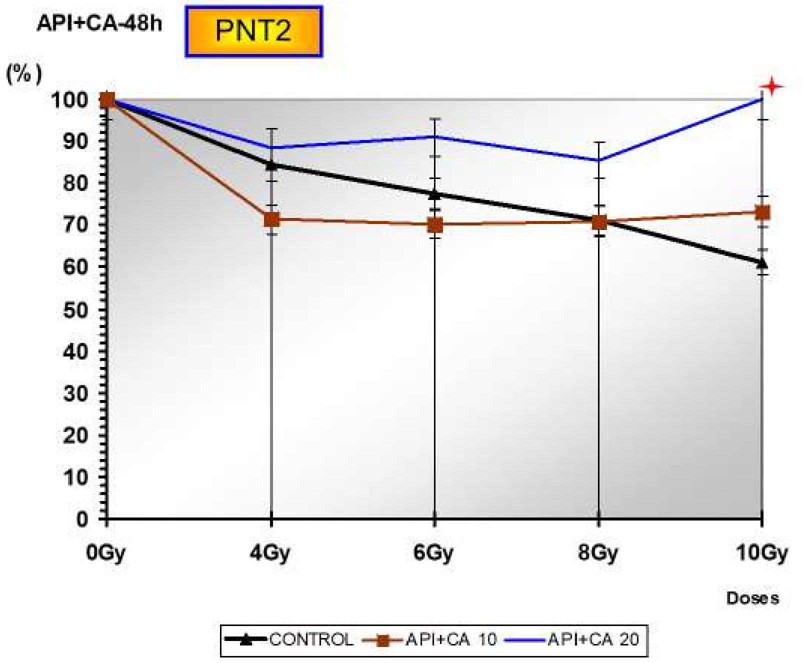

Figure S12. Cell survival curves of normal human prostate epithelial PNT2 cell cultures treated with 10 μM and 20 μM mixtures of (API + CA) and exposed to different doses of X-rays evaluated after 48 hours of incubation ((*): (p<0.001) versus irradiated control cell cultures).

When cultured cells were exposed to the highest experimental radiation dose of 10 Gy and incubated for 48 hours, the PF obtained with samples treated with apigenin and carnosic acid mixture was 30.9% with the 10 μM mixture and 100% when the 20 μM

mixture was used. The DRF obtained in the samples treated with the equimolar mixtures at the highest experimental radiation dose of 10 Gy was 1.25 for the 10 µM mixture, however, at this same irradiation dose, a corresponding value could not directly be evaluated when the 20 µM mixture was used.

- MIXTURES Pycnanthus angolensis Warb Seed Extract (PASE) AND CARNOSIC ACID (PASE + CA)

The co-administration of PASE [34] and CA at concentrations of 25 µl of 25µM PASE and 20µl of 20 µM CA respectively, did not lead to a decrease in the percentage of proliferating PNT2 cells when compared to that obtained from control PNT2 cell cultures (Figure S10).

After 24-hours of incubation, PNT2 cell cultures co-treated with PASE and CA (25 µl+ 20 µl of 10 µM respectively), demonstrated an increase in cellular survival, with respect to irradiated control PNT2 cell cultures. The percentage cell survival of test cell culture was 27.3% higher than what was measured for control cell cultures at the highest experimental dose of 10 Gy and the test substance concentration used. At the concentration of PASE and CARN studied, a significant increase in PNT2 cell survival was observed compared with irradiated control cell cultures (p<0.001) (Table S9; Figure S13)

Table S9. Percentage cell survival (%) of normal human prostate epithelial PNT2 cell cultures treated with (PASE + CA) and exposed to different doses of X-rays evaluated after 24 hours of incubation.

Radiation doses	0 Gy	4 Gy	6 Gy	8 Gy	10 Gy
Controls	100.0 ± 5.8	91.0 ± 7.1	86.2 ± 7.2	79.4 ± 5.4	69.2 ± 5.1
PASE and CA (20 µl+10 µM)	100.0 ± 7.8	93.0 ± 9.4	91.3 ± 9.8	90.3 ± 7.5	96.5 ± 7*

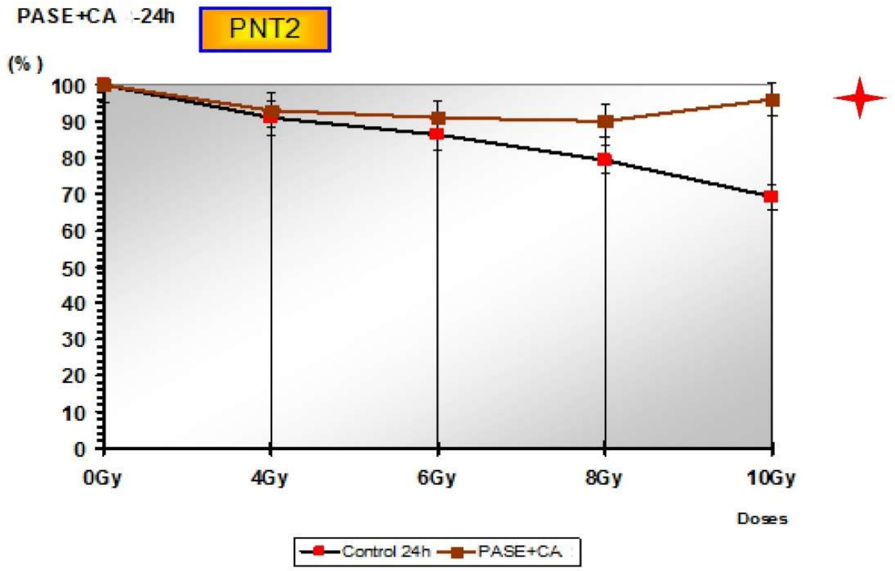

Figure S13. Cell survival curves of normal human prostate epithelial PNT2 cell cultures treated with (PASE + CA) and exposed to different doses of X-rays evaluated after 24 hours of incubation ((*): (p<0.001) versus irradiated control cell cultures.

The **Protection Factor** (**PF**) obtained from 24-hour PNT2 cell cultures co-treated with PASE and CARN and exposed to the highest experimental radiation dose (10 Gy) was 88.6%. The **Dose Reduction Factor** (**DRF**) obtained from PNT2 cell cultures co-treated with PASE and CA, exposed to the highest experimental radiation dose of 10 Gy and incubated for 24 hours was 5.8.

After 48 hours of incubation, PNT2 cell cultures co-treated with PASE and CARN at the studied concentrations also showed an improved cellular survival rate compared with irradiated PNT2 control cell cultures. The survival rate in the test cell cultures was 12% higher than that obtained in the irradiated control PNT2 cell cultures at the highest experimental radiation dose (10 Gy) and the test substance concentrations used ($p<0.01$) (Table S10; Figure S14).

Table S10. Percentage cell survival (%) of normal human prostate epithelial PNT2 cell cultures treated with (PASE + CA) and exposed to different doses of X-rays evaluated after 48 hours of incubation.

Radiation doses	0 Gy	4 Gy	6 Gy	8 Gy	10 Gy
Controls	100.0 ± 4.5	84.5 ± 8.2	77.3 ± 7.4	71.1 ± 5.6	61.2 ± 5.2
PASE and CA (25 μl + 10 μM)	100.0 ± 8.2	90.0 ± 7.4	81.2 ± 9.1	75.8 ± 7.5	73.2 ± 8.9*

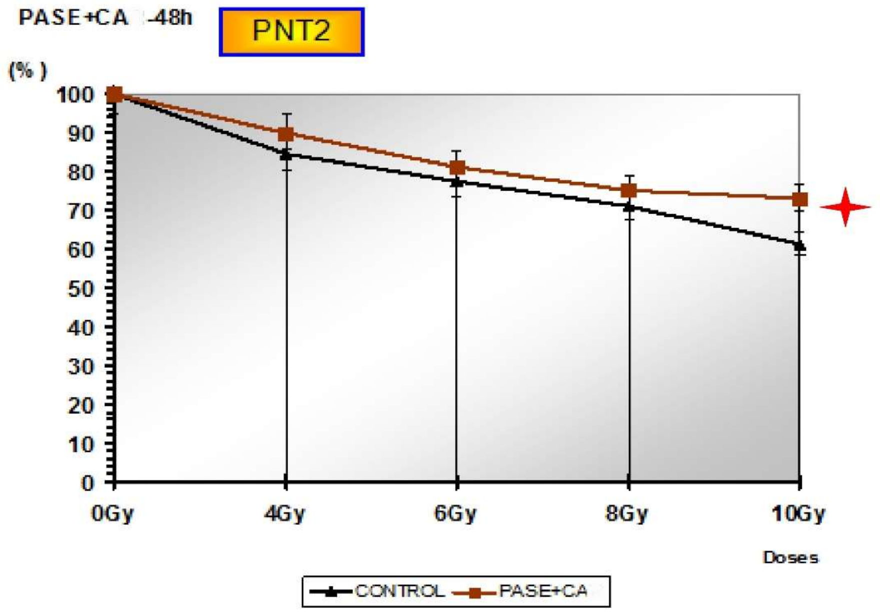

Figure S14. Cell survival curves of normal human prostate epithelial PNT2 cell cultures treated with (PASE + CA) and exposed to different doses of X-rays evaluated after 48 hours of incubation ((*): ($p<0.001$) versus irradiated control cell cultures.

The **Protection Factor** (**PF**) obtained in the PNT2 cell culture samples treated with PASE and CA and exposed to the highest experimental radiation dose (10 Gy) was 30.9 when the cells were incubated for 48 hours. The **Dose Reduction Factor** (**DRF**) obtained in the PNT2 cell culture samples treated with PASE and CA and exposed to the highest dose (10 Gy) used in this study was 1.25.

Using the proposed parameters (concentration, X-ray dose and incubation period), these results confirm that CA maintains and/or enhances its radioprotective effects when administered with other antioxidant substances in PNT2 cells.

4. B) B16F10 CELLS

MIXTURES APIGENIN AND CARNOSIC ACID (API + CA)

Admixtures of apigenin and carnosic acid (API + CA) at concentrations of 10 μM (10 μM API + 10 μM CARN) and 20 μM (20 μM API + 20 μM CARN) were administered to cell cultures and their effect on radioprotection assessed.

After 24 hours of B16F10 cell incubation with co-treatment of apigenin and carnosic acid at (10 μM and 20 μM) concentrations, and exposure of cell cultures to the highest experimental radiation dose (10 Gy), a decrease in cell survival was shown compared with irradiated control cell cultures. A decrease of 46.9% was observed for the 10μM combination while the 20μM combination produced only 3% decrease in cell. No radioprotective effect was determined for this mixture (Table M29; Figure M35), however, contrarily, the results obtained showed a radiosensitizing effect of the 10μM (API + CARN) mixture on B16F10 melanoma cells (**p<0.001**) (Table S11; Figure S15) similar to the effect shown by administration of 10 μM CA alone.

Table S11. Percentage cell survival (%) of B16F10 cell cultures treated with 10 μM and 20 μM mixtures of (API + CARN) and exposed to different doses of X-rays evaluated after 24 hours of incubation.

Radiation doses	0 Gy	4 Gy	6 Gy	8 Gy	10 Gy
Controls	100.0 ± 9.5	86.7 ± 7.4	80.6 ± 8.2	73.2 ± 6.8	66.9 ± 7.7
10 μM (API + CA)	100.0 ± 6.9	79.0 ± 9.2	45.2 ± 7.8	34.3 ± 8.4	20.0 ± 9.1*
20 μM (API + CA)	100.0 ± 8.1	92.8 ± 7.9	90.9 ± 5.3	77.0 ± 6.2	63.9 ± 7.5

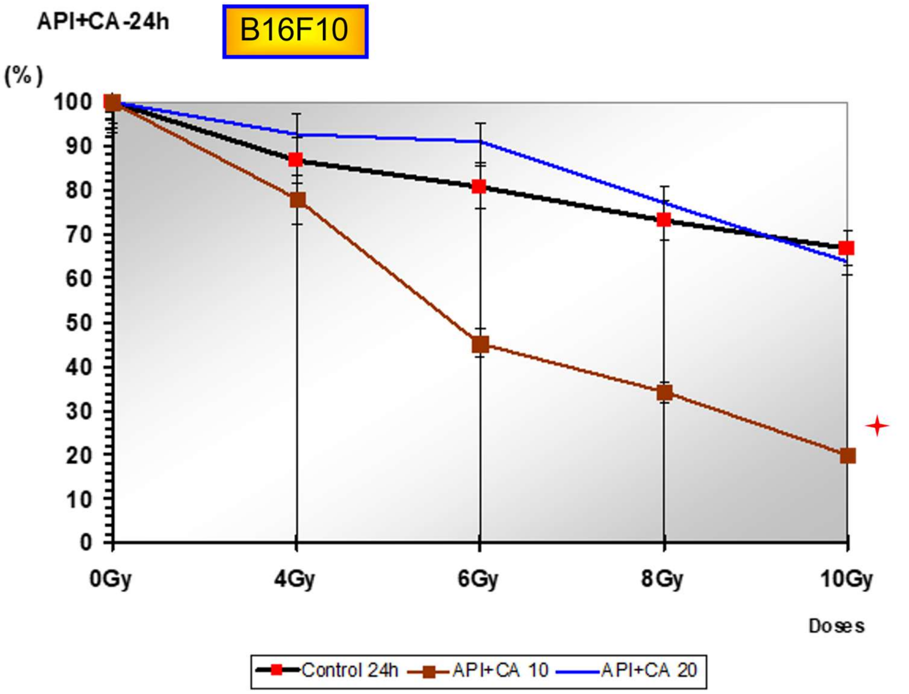

Figure S15. Cell survival curves of B16F10 cell cultures treated with 10 μM and 20 μM mixtures of (API + CA) and exposed to different doses of X-rays evaluated after 24 hours of incubation. ((*): (p<0.001) versus irradiated control cell cultures).

24-hour B16F10 cell cultures simultaneously treated with 10 μM and 20 μM each of carnosic acid and apigenin and exposed to a radiation dose of 10 Gy produced a radiation **Protection Factor** (**PF**) of zero, i.e. no radiation protection capacity for both combinations of the test substances. It was not possible to obtain a **Dose Reduction Factor** (**DRF**) for the 24-hour B16F10 cell cultures simultaneously treated with any of the combinations (10 μM or 20 μM) of carnosic acid and apigenin mixture and exposed to the highest dose of radiation (10 Gy) since neither solutions showed any radioprotection.

The 48-hour B16F10 cell cultures simultaneously treated with both combinations of apigenin and carnosic acid (10 μM and 20 μM) and exposed to the highest experimental

radiation dose (10 Gy), showed a decrease in cell survival with respect to the irradiated control cell cultures. Cell proliferation in cultures exposed to the 10 μM combination decreased by 8.3% while those exposed to the 20 μM combination produced a decrease of 45.8% in cell survival. No radioprotective effect was established for this mixture (Table S12; Figure S16). However, conversely the results obtained showed a radiosensitizing effect of the 20μM (API + CA) mixture on B16F10 melanoma cells (**p <0.001).**

Table S12. Percentage cell survival (%) of B16F10 cell cultures treated with 10 μM and 20 μM mixtures of (API + CA) and exposed to different doses of X-rays evaluated after 48 hours of incubation.

Radiation doses	0 Gy	4 Gy	6 Gy	8 Gy	10 Gy
Controls	100.0 ± 0	74.7 ± 8.3	72.0 ± 6.8	66.0 ± 7.4	59.0 ± 5.9
10 μM (API + CARN)	100.0 ± 7.9	98.5 ± 8.1	81.3 ± 9.2	64.9 ± 7.5	50.7 ± 8.3
20 μM (API + CARN)	100.0 ± 7.7	91.8 ± 6.9	61.9 ± 5.3	24.0 ± 7.4	13.12 ± 12*

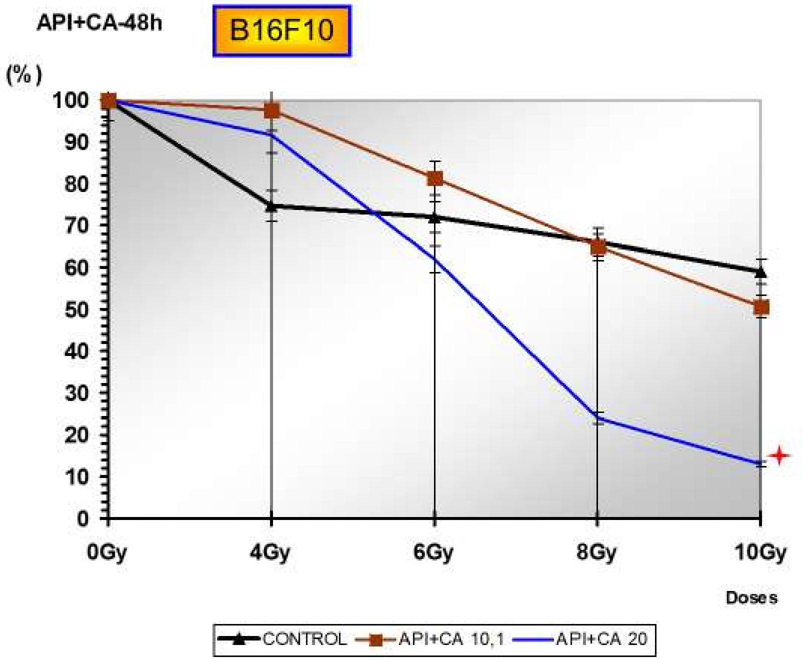

Figure S16. Cell survival curves of B16F10 cell cultures treated with 10 μM and 20 μM mixtures of (API + CA) and exposed to different doses of X-rays evaluated after 48 hours of incubation. ((*): (p<0.001) versus irradiated control cell cultures).

A **Protection Factor (PF)** of zero was obtained when 48-hour B16F10 cell cultures simultaneously treated with both combinations of carnosic acid apigenin (10 μM, as for the 20 μM) and irradiated with the highest experimental radiation dose (10 Gy) i.e., no protective capacity was shown by either test substance combination (10 μM or 20 μM). It was not possible to evaluate a **Dose Reduction Factor** (**DRF**) for either of the 48-hour B16F10 cell cultures co-treated with the two combinations of apigenin and carnosic acid (10 μM and 20 μM) and exposed to the highest radiation dose used in this study (10 Gy). This was so because the radiation dose that produces the same effects could not be determined without a radioprotective property of the test compounds.

- PASE AND CARNOSIC ACID MIXTURE (PASE + CA)

In 24-hour irradiated B16F10 cell cultures simultaneously exposed to both PASE and carnosic acid (25 μl of 25 μM PASE + 20 μl of 10 μM CA the highest experimental radiation

dose (10 Gy) used showed a decrease in cell survival, compared to irradiated control cells, decreasing by 46.9% when referenced with control cell culture survival values. No radioprotective effect was determined. The results obtained demonstrates a radiosensitizing effect of the simultaneous administration (PASE + CARN) on B16F10 melanoma cells **(p <0.001)** (Table S13, Figure S17).

Table S13. Percentage cell survival (%) of B16F10 cell cultures treated with (PASE + CARN) and exposed to different doses of X-rays evaluated after 24 hours of incubation.

Radiation doses	0 Gy	4 Gy	6 Gy	8 Gy	10 Gy
Controls	100.0 ± 5.8	86.7 ± 9.1	80.6 ± 7.7	73.2 ± 7.5	66.9 ± 7.5
25 μl of 25 μM PASE + 10 μl of 10 μM CARN	100.0 ± 7.6	79.0 ±7.8	45.2± 5.8	34.3 ± 9.4	20.0 ± 5*

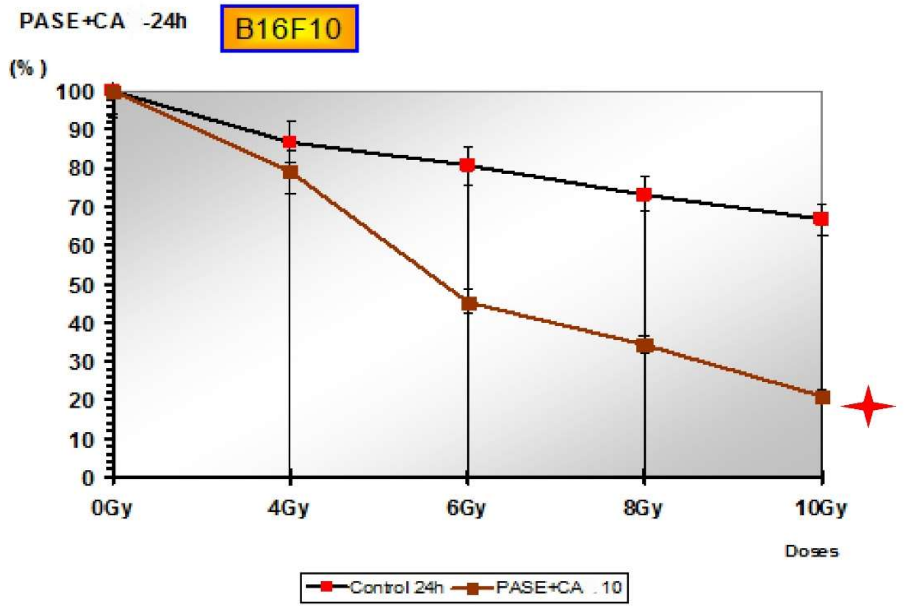

Figure S17. Cell survival curves of B16F10 cell cultures treated with (PASE + CARN) and exposed to different doses of X-rays evaluated after 24 hours of incubation. ((*): (p<0.001) versus irradiated control cell cultures).

The **Protection Factor** (**PF**) obtained from 24-hour B16F10 cultures treated with PASE and CARN after exposure to the highest irradiated dose (10 Gy) used in this study was zero (0) i.e., it lacked radioprotective capacity. It was however, not possible to compute the **Dose Reduction Factor** (**DRF**) for the 24-hours B16F10 cell cultures treated with PASE and carnosic acid and exposed to the highest radiation dose of 10 Gy since it had no radioprotective capacity. This implies that it presents no dose-lowering effect.

In 48-hour B16F10 cell cultures co-treated with PASE and CARN at (25 μl of 25 μM PASE + 20 μl of 10 μM CARN) respectively and exposed to the highest experimental radiation dose (10 Gy), there was a decrease in percentage cell survival when compared with irradiated control cell cultures. The percentage cell survival of the irradiated control cohort was 8.3% superior to cell cultures that were co-treated with PASE and carnosic acid.

Table S14. Percentage cell survival (%) of B16F10 cell cultures treated with (PASE + CARN) and exposed to different doses of X-rays evaluated after 48 hours of incubation.

Radiation doses	0 Gy	4 Gy	6 Gy	8 Gy	10 Gy

Controls	100.0 ±	74.7 ±	72.0 ±	66.0 ±	59.0 ±
25 μl of 25 μM PASE + 20 μl of 10 μM CARN	100.0 ±	98.5 ±	81.3 ±	64.9 ±	50.7 ± 4

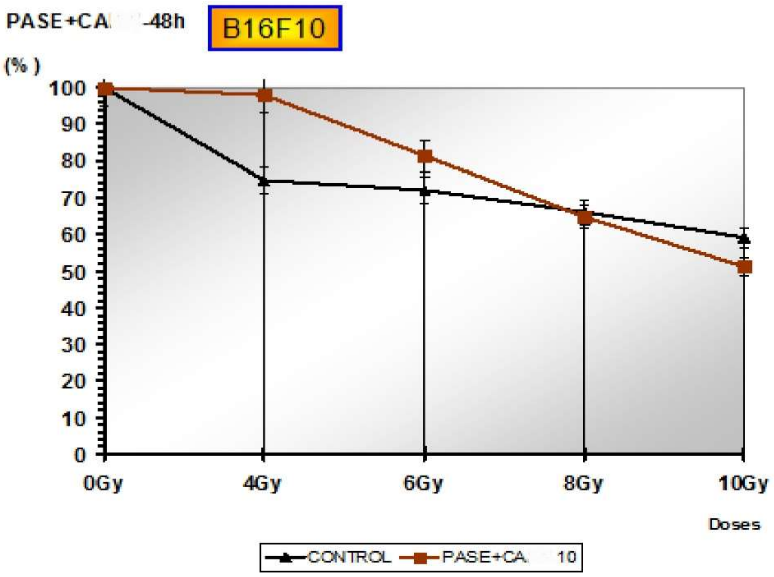

Figure S18. Cell survival curves of B16F10 cell cultures treated with (PASE + CARN) and exposed to different doses of X-rays evaluated after 48 hours of incubation. ((*): ($p<0.001$) versus irradiated control cell cultures).

The **Protection Factor** (**PF**) obtained in the 48 hours B16F10 cell culture samples treated with PASE and carnosic acid and exposed to the highest experimental radiation dose (10 Gy) was zero i.e., it had no protective capacity. It was not possible to evaluate the **Dose Reduction Factor** (**DRF**) in the samples treated with PASE and carnosic acid and exposed to the highest dose used in this study (10 Gy) since it did not show any radioprotective capacity. This suggests that the treatment presents no dose-lowering effect.

With the proposed parameters (CA concentration, X-ray dose and incubation period), these results confirm that CA maintains its paradoxical radiosensitizing effects even when administered together with other antioxidant substances in PNT2 cells.

Process of involving the determination of selected cell culture parameters in the manuscript to establish cell survival using the Clonogenic assay.

The clonogenic assay was performed using four different radiation doses in the following descending order (10Gy, 8Gy, 6Gy and 4Gy) because the limiting factor of the analysis was the presence of colonies in the Ci group of PNT2 cells that permitted the counting of the colonies formed. The highest dose of radiation tested that allowed this objective was 4 Gy.
